# Enhancing strawberry salt stress tolerance: morphophysiological responses to different silicon rates and sources

**DOI:** 10.1038/s41598-025-34089-x

**Published:** 2025-12-28

**Authors:** Peyman Kashani, Ali Khanmirzaei, Seied Mehdi Miri, Shekoofeh Rezaei, Khodadad Mostafavi

**Affiliations:** 1 Department of Horticultural Science, Ka. C., Islamic Azad University, Karaj, Iran; 2 Department of Soil Science, Ka. C., Islamic Azad University, Karaj, Iran; 3 Department of Agronomy and Plant Breeding, Ka. C., Islamic Azad University, Karaj, Iran

**Keywords:** Abiotic stress, Chlorophyll, Fertilizers, Plant physiology, Spectral analysis, Silicon, Environmental sciences, Plant sciences

## Abstract

Salinity stress is a critical environmental factor that significantly reduces strawberry productivity. The aims of the present study were to evaluate the efficacy of four foliar-applied silicon (Si) sources such as nano-silica, organic silica, potassium silicate, and stabilized silicic acid and rates including zero, 10, and 30 mg Si L^− 1^ on morphological properties of strawberries in two salinity levels (0 and 50 mM NaCl). A factorial experiment in a randomized complete block design with three replications was conducted. Key physiological parameters such as chlorophyll contents, free proline, and fruit yield were measured. Results showed that stabilized silicic acid and potassium silicate at 30 mg L^− 1^ significantly enhanced photosynthetic pigment concentrations, reduced oxidative stress, and improved yield under salt stress. Additionally, Vis-NIR data coupled with partial least squares regression (PLSR) moderately predicted leaf Si content under saline conditions (R^2^ = 0.453 and MSE = 1.94). These findings highlight the potential of Si fertilization to mitigate salinity impacts in strawberry, while precision spectral tools such as Vis–NIR spectroscopy can support non-destructive monitoring of Si-induced physiological responses.

## Introduction

Strawberry (Fragaria × ananassa) is a globally important berry fruit crop, and it is grown at a large scale. However, strawberries are susceptible to salt-induced morpho-physiological stress highlighted in several studies^1,2^ and they are considered one of the most salt-sensitive horticultural crops and even low salinity levels can reduce strawberry yield and biomass^3^. Strawberries are considered highly sensitive to salinity, with a threshold of approximately 0.7 dS m⁻¹, beyond which yield declines by about 33% for each additional unit increase in salinity^4^. More recent studies confirm that even moderate salinity levels (2–4 dS m⁻¹) significantly reduce strawberry yield and fruit quality^12^. Of soluble salts, sodium chloride (NaCl) is the most common and injurious that produce severe adverse effects on plant growth and fruit yields^5^. Exposure of strawberries to NaCl-induced salinity stress has been linked to increased leaf necrosis and accelerated senescence, promoted lipid peroxidation and significantly declined photosynthetic efficiency^6–8^.

Soil salinization is mostly controlled by multiple interrelated factors including geology (e.g., parent material rich in soluble salts), soil chemical properties (cation exchange capacity, gypsum and carbonate contents), the hydrological regime (groundwater depth, irrigation practices, and drainage conditions), and climatic variables such as high evapotranspiration relative to precipitation^9^. The phenomenon usually occurs where evapotranspiration rates are significantly greater than rainfall and the salts accumulate in the soil profile^10^. The salts are redistributed vertically and horizontally by water flow^11^. Climate change has increased soil salinization to become one of the most environmental challenges specifically in arid and semi-arid regions^12,13^. The issue is common in different regions of the world such as China^14^, United States^15^, Iran^9,10,16^, and Turkey^17^. Therefore, evaluating and finding some methodology to decrease the soil salinity stresses is essential.

Silicon (Si), as a beneficial nutrient, has emerged as a potential element for improving plant resilience under stressful environmental conditions^18^. Si enhances plant tolerance to salinity through several physiological mechanisms such as improving water-use efficiency, reducing sodium uptake, enhancing antioxidant defense systems, and stabilizing membrane integrity^19^. Numerous studies have demonstrated the efficacy of Si in enhancing plant resistance to abiotic stresses like drought, salinity, and heavy metal toxicity. For instance, Zhu and Gong^20^ emphasized that Si improves salt and drought tolerance in various crops through physiological and biochemical regulation. Zhang et al.^21^ reported Si-induced improvements in legumes under saline conditions. Studies by Simaei et al.^22^ on cucumber and Soundararajan et al.^23^ on rose also demonstrated the positive influence of Si on antioxidant enzyme regulation and osmotic adjustment under salinity. In strawberries, Park et al.^24^ observed that both foliar and sub-irrigational application of Si mitigated the negative effects of salinity during vegetative stage. Moreover, Ouellette et al.^25^ confirmed that Si application increased chlorophyll concentration and reduced sodium accumulation in strawberry plants grown under field and high tunnel conditions. Despite its abundance in soils, Si availability to plants is limited depending on soil composition and environmental conditions^26^.

Although some studies have explored the effects of Si under saline conditions, only a limited number have investigated strawberries, and most of these have focused on single Si sources or soil-applied treatments. For instance, previous work has largely emphasized potassium silicate or bulk silica amendments with little comparative evidence across multiple foliar-applied forms. Different Si fertilizers vary in chemical composition, solubility, and uptake pathways, which can significantly influence their effectiveness in plants^21–26^. Potassium silicate is a widely used soluble Si source, supplying both potassium and silicic acid, but may precipitate under certain conditions. Stabilized silicic acid delivers Si in a monomeric, highly bioavailable form that is readily absorbed by leaves. Nano-silica consists of ultrafine particles with high surface reactivity, potentially enhancing foliar absorption, though its mobility inside the plant remains under study. Organic silica formulations are often designed to improve Si stability and penetration through leaf cuticles. Because strawberries are considered poor Si accumulators, differences in solubility and plant uptake among these sources may significantly affect their capacity to mitigate salt stress. Hence, a clear understanding of which foliar-applied Si sources are most effective for strawberries is still lacking. Furthermore, no studies have integrated spectral monitoring tools to non-destructively assess Si-induced physiological changes in strawberries under salinity. This creates a critical knowledge gap regarding both the comparative efficacy of multiple Si fertilizers and the potential of precision spectral methods to track Si-related improvements. This study addresses that gap by evaluating the effectiveness of Si sources and rates under salinity stress. It was hypothesized that Si sources with higher solubility and foliar bioavailability can produce greater improvements in photosynthetic pigments, osmotic regulation, and yield and that Vis–NIR spectral data would reflect these Si-mediated physiological changes. Hence, the present study aimed to (i) investigate the morphophysiological responses of strawberry plants to different foliar-applied Si sources and rates under salinity stress and (ii) monitor their influences on physiological and biochemical parameters by using Vis-NIR data.

## Materials and methods

### Study area

**Fig. 1:**
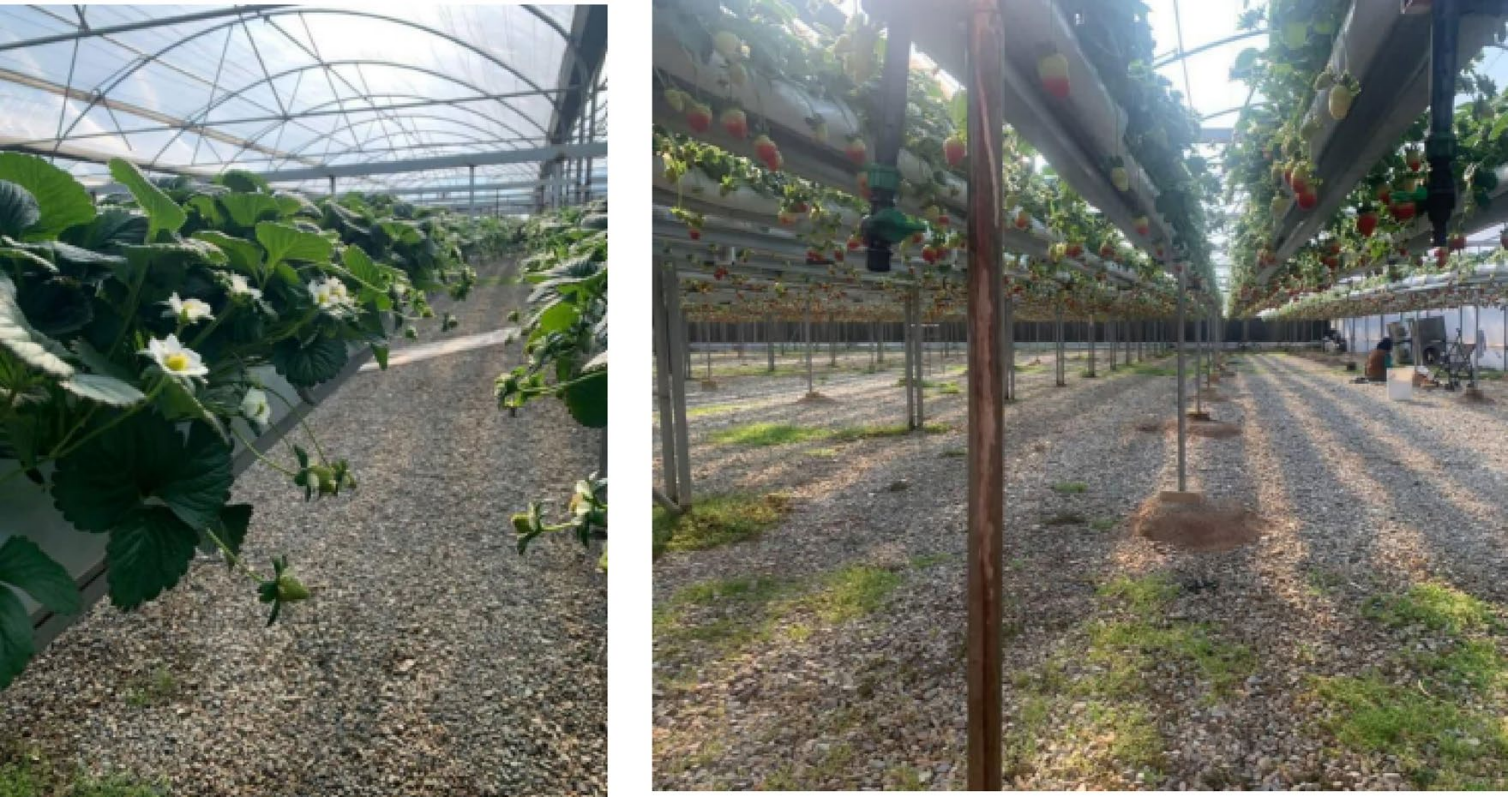
Two stages of strawberry growth in the greenhouse.

The experiments were conducted in a controlled greenhouse environment at the Islamic Azad University, Karaj Branch, Alborz Province, Iran (Fig. 1). The strawberry cultivar for this study was *Camarosa*. The commercial strawberry cultivar Fragaria × ananassa cv. Camarosa was used in this study, and its identity was confirmed by the Principal Investigator (Dr. Ali Khanmirzaei) at the time of planting.

### Experimental design

Plants were cultivated in a soilless system using containers filled with cocopeat and perlite (1:1 v/v). The cocopeat–perlite substrate used for plant cultivation had an initial electrical conductivity (EC) of 0.4 dS m^−1^ and a pH of 5.8 prior to transplanting. At the end of the experiment, substrate EC increased to 0.9 dS m^−1^ in the control treatment and up to 3.5 dS m^−1^ under the 50 mM NaCl salinity treatment, while pH remained relatively stable between 5.7 and 6.0 across treatments. This medium provided high water-holding capacity and good aeration. Plants were spaced at 80 cm between rows and 25 cm within rows. Irrigation was supplied through a drip system, delivering water and nutrients to maintain optimal substrate moisture throughout the experiment.

Foliar applications of Si were initiated two weeks after transplanting and continued at 10-day intervals, with a total of six sprayings applied until the end of the experiment. Silicon treatments were applied at 0, 10, and 30 mg L⁻¹, and solutions were prepared freshly before each application to have consistency. Si treatments were applied via foliar spray using a hand-held sprayer until leaf surfaces were fully covered.

A factorial experiment arranged in a randomized complete block design (RCBD) was conducted with three replications. Experimental factors included four Si sources (nano-silica, organic silica, potassium silicate, and stabilized silicic acid), three Si application rates (Zero, 10, and 30 mg L^− 1^), and in two salinity levels (Zero and 50 mM NaCl) (Table 1). Treatments were randomly assigned within each block to minimize positional effects and ensure unbiased comparisons.

Si treatments were applied via foliar spray using a hand-held sprayer until leaf surfaces were fully covered. Salinity stress was imposed by adding NaCl to the irrigation water to reach a concentration of 50 mM, while control plants received distilled water without supplemental Si or NaCl. Sampling for chlorophyll content, free proline, Si concentration, and yield was performed 60 days after transplanting at the peak fruiting stage.

**Table 1:** Combinations of different Si resources and rates in various salinity stress.

Salinity (mM)	Si sources	Si rate (mg L^− 1^)
Zero	Nano silica	Zero, 10, and 30
	Organic silica	Zero, 10, and 30
	Potassium silicate	Zero, 10, and 30
	Stabilized silicic acid	Zero, 10, and 30
50	Nano silica	Zero, 10, and 30
	Organic silica	Zero, 10, and 30
	Potassium silicate	Zero, 10, and 30
	Stabilized silicic acid	Zero, 10, and 30

### Mineral analysis

Shoot samples were dried at 70 °C for 48 h and then powdered into fine powder. To achieve this, 500 mg of dry plant material was placed in each of 12 Teflon microwave digestion tubes and wetted with 3 mL of nitric acid and 1 mL of Hydrofluoric acid following the procedure described by Ma and Takahashi^47^. The tubes were closed and left to stand for 10 min for adequate wetting. Next, the samples were then digested in the temperature 120–150 ^◦^C. Finally, Si content was analyzed by Inductively Coupled Plasma Optical Emission Spectrometry (ICP-OES).

### Phenotype measurement

Free proline content in the leaves of strawberry was estimated according to the method of Bates et al.^27^. The fresh leaf tissue (500 mg) was homogenized in 5 mL of 3% sulfosalicylic acid using a mortar and pestle. The homogenate was centrifuged at 3500 × g for 10 min using a refrigerated centrifuge (Eppendorf 5810 R, Germany), and clear supernatant was used for analysis. To each sample, 2 mL of acid ninhydrin solution (obtained by dissolving 1.25 g ninhydrin in 30 mL glacial acetic acid and 20 mL of 6 M phosphoric acid with gentle heating) and 2 mL glacial acetic acid were added. The solution was incubated for 60 min in a 100 °C water bath and incubation was terminated by placing the tubes in an ice bath. The reaction mixture was extracted with 4 mL of toluene and agitated well with a test tube stirrer for 15–20 s. The toluene phase was read for absorbance at 520 nm using a UV–Vis spectrophotometer (Shimadzu UV-1800, Japan). Free proline content was quantified with respect to a standard curve with L-proline and expressed as µmol g^− 1^ fresh weight.

Leaf pigment parameters, including chlorophyll, were measured following the method of Richardson et al.^28^. To start with, 500 mg of leaf tissue was placed in each tube containing 50 mL of 80% acetone solution. Samples were homogenized properly, and the developed extract was centrifuged at 3000 × g for 10 min. The supernatant absorbance was read then at specific wavelengths: 663 nm for chlorophyll a and 645 nm for chlorophyll b using the Shimadzu UV-1800 spectrophotometer. The pigment concentrations were then computed using standard formulas^28^.

1 $$\:Chl\:a(g/lit)\:=0.0127A663\:-0.0269A645$$

2 $$\:Chl\:b(g/lit)\:=0.0029A663\:-0.00468A645$$

At the end, total dry biomass and fruit yield per plant was measured.

### Statistical analysis

All data obtained were analyzed using a three-way analysis of variance (ANOVA). Prior to ANOVA, datasets were tested for normality using the Shapiro–Wilk test and for homogeneity of variance using Levene’s test in SAS (version 9.4, Cary, NC, USA). Means comparison was performed using the least significant difference (LSD) test at *p* ≤ 0.05.

### VNIR data

VNIR spectral data were collected under darkroom conditions using an ASD FieldSpec3 spectrometer across the wavelength range of 350–1100 nm. The objective was to analyze plant spectral properties. For each plant sample, 15 spectra were recorded and averaged to reduce variation and enhance consistency. The raw reflectance data (R) were converted to values of absorbance using the equation A = log(1/R). To minimize noise and baseline drift, a Savitzky–Golay smoothing filter (second-order polynomial, window size = 15 points) was applied prior to analysis. Outlier spectra were identified and removed before calibration. This was able to suppress baseline drifts and lower noise in spectral signals^29^. Partial Least Squares Regression (PLSR)^30^ was then employed to model Si concentration in plants. The model focused on finding relationships between the most sensitive spectral bands and Si content. Model performance was evaluated using full cross-validation (leave-one-out) to reduce the risk of overfitting^31^. The data processing was conducted based on R software and Python (version 3.8.5), while the PLSR modeling itself was carried out based on the Unscrambler X 10.3 software^32,33^.

The performance of the model was validated by various main statistical indicators such as coefficient of determination (R²)^11^ root mean square error (RMSE)^34^, mean error (ME), and ratio of predicted deviation (RPD)^35^.

## Results

### Chlorophyl a, b, free proline, Si concentration, and yield

Table 2 illustrates the comparison of mean values for chlorophyll a and b contents among strawberry plants treated with different Si fertilizers sources, rates, and different salinity levels. Under the fertilizer Si sources applied, organic silica and stabilized silicic acid produced the highest value of chlorophyll a followed closely by nano silica and potassium silicate (Table 2). For chlorophyll b, organic silica yielded the highest value (Table 2). Si application rates were 0 to 30 mg L⁻¹ and caused increasing contents of chlorophyll a, ranging from 0.95 to 1.06 mg g^− 1^ with a 11.6% increase (Table 2). However, chlorophyll b remained relatively unchanged across rates. Salinity stress decreased chlorophyll a, with a mean of 0.88 at 50 mM salinity and a 23% decrease in comparison with non-saline conditions (Table 2). Chlorophyll b was also reduced under salinity (0.23 mg g^− 1^) compared to the non-saline treatment (0.25 mg g^−1^) (Table 2). These results showed that the most suitable Si sources for maintaining leaf chlorophyll levels are stabilized silicic acid and organic silica (Table 2).

The mean free proline contents in strawberry plants under different situations are presented in Table 2. Among the Si fertilizers, potassium silicate produced the highest free proline content (0.38 mg g^− 1^) and stabilized silicic acid showed the lowest value (0.31 mg g^− 1^) (Table 2). Salinity had a marked effect on free proline accumulation under 50 mM salinity showing significantly higher proline levels with a 73% increase (Table 2). These results suggest that salinity stress strongly induces free proline accumulation in strawberry plants.

**Table 2:** Mean comparison of chlorophyll a and b strawberry under different treatments.

Treatments	Chlorophyll a (mg g^− 1^)	Chlorophyll b (mg g^− 1^)	Free proline (µmol g^− 1^)	Si concentration (mg g^− 1^)	Yield (g bag^− 1^)
Si fertilizer type					
Potassium silicate	0.97^a^	0.21^b^	0.38^a^	1.520^c^	950.29^a^
Nano silica	0.98^a^	0.23^ab^	0.37^a^	1.340^d^	754.19^b^
Organic silica	1.04^a^	0.27^a^	0.37^a^	1.628^b^	945.77^a^
Stabilized silicic acid	1.04^a^	0.25^a^	0.31^a^	1.756^a^	926.14^a^
Si rates (mg L^− 1^)					
Zero	0.95^b^	0.24^a^	0.38^a^	0.931^c^	877.77^a^
10	1.02^ab^	0.24^a^	0.35^a^	1.654^b^	885.46^a^
30	1.06^a^	0.25^a^	0.34^a^	2.098^a^	919.06^a^
Salinity level (mM)					
Zero	1.14^a^	0.25^a^	0.26^b^	1.534^b^	1116.61^a^
50	0.88^b^	0.23^b^	0.45^a^	1.588^a^	671.59^b^

The same letters (e.g., a, b, and c) are not significantly different at *p* ≤ 0.05 (LSD test). In each column, means followed by the same letter are not significantly different at *p* ≤ 0.05.

### Analysis of interactions

#### Chlorophyll b

**Fig. 2:**
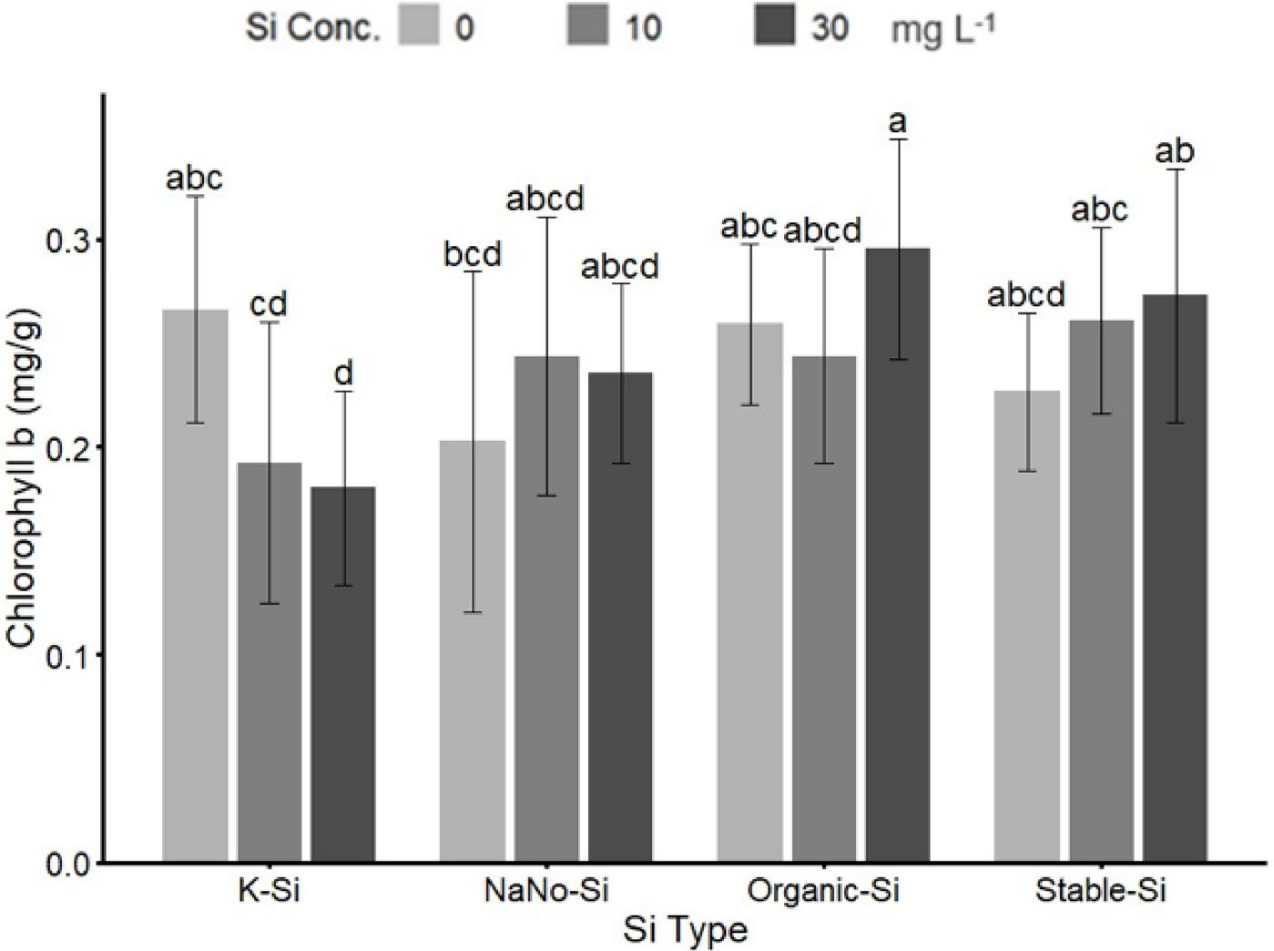
Interaction effect of Si fertilizer type (K-Si, NaNo-Si, Organic-Si), and Stable-Si) and Si concentrations (0, 10, and 30 mg L^− 1^) on chlorophyll b. Bars sharing the same letter are not significantly different at *p* ≤ 0.05 (LSD test).

The analysis of chlorophyll b contents in strawberry plants under different Si treatments showed significant effects of both Si type and application rate (Fig. 2). Among the tested fertilizers, organic silicon at 30 mg L^−1^ resulted in the highest chlorophyll b concentration (0.295 mg g^−1^), which was approximately 39% higher. In contrast, the control treatment (potassium silicate at zero Si) showed the lowest chlorophyll b levels (0.180 mg g^−1^) (Fig. 2). The interaction between Si fertilizer type and concentration was evident, with organic silicon consistently enhancing chlorophyll b levels across increasing Si concentrations. The potassium silicate and stabilized silicic acid diminished returns at higher rates. Nano-silica treatments produced increases in chlorophyll b content. However, it was less effective compared to organic silicon. Under salinity stress (50 mM), chlorophyll contents and overall plant performance declined. However, the application of Si, specifically organic silicon, helped mitigate these negative effects.

#### Si concentration

**Fig. 3:**
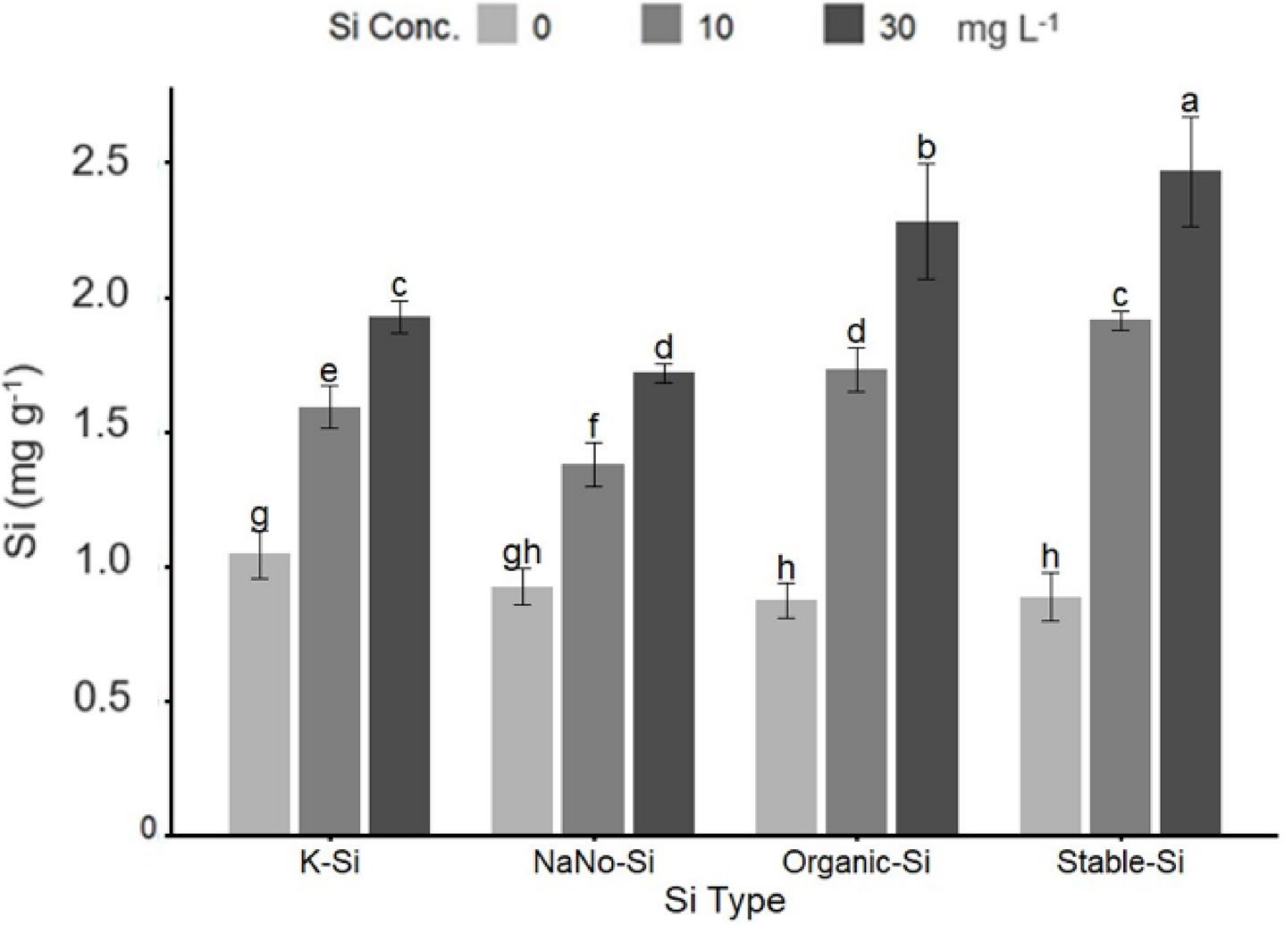
Effect of different types (K-Si, NaNo-Si, Organic-Si) and rates (0, 10, and 30 mg L^−1^) of Si fertilizer on Si concentration on Strawberry leaves. Bars sharing the same letter are not significantly different at *p* ≤ 0.05 (LSD test).

Figure 3 indicates the application of Si fertilizers significantly affected the Si concentration in strawberry leaves. Among the treatments, stable silicon at 30 mg L^−1^ produced the highest Si concentration, more than double (≈ 125% increase) (Fig. 3). Organic silica at 30 mg L^−1^ and potassium silicate at the same concentration also resulted in high Si accumulation, though slightly lower than stable silicon (Fig. 3). In contrast, the lowest leaf Si concentrations were observed in plants treated with nano silica at zero mg L^− 1^ (control) followed by organic silica and stable silicon at zero mg L^−1^ (Fig. 3). Increasing the Si concentration in the fertilizer treatments from zero to 30 mg L^−1^ consistently led to higher Si uptake by the leaves at all Si sources (Fig. 3).

### Analysis of yield

#### Effect of different rates and types of Si fertilizers

**Fig. 4:**
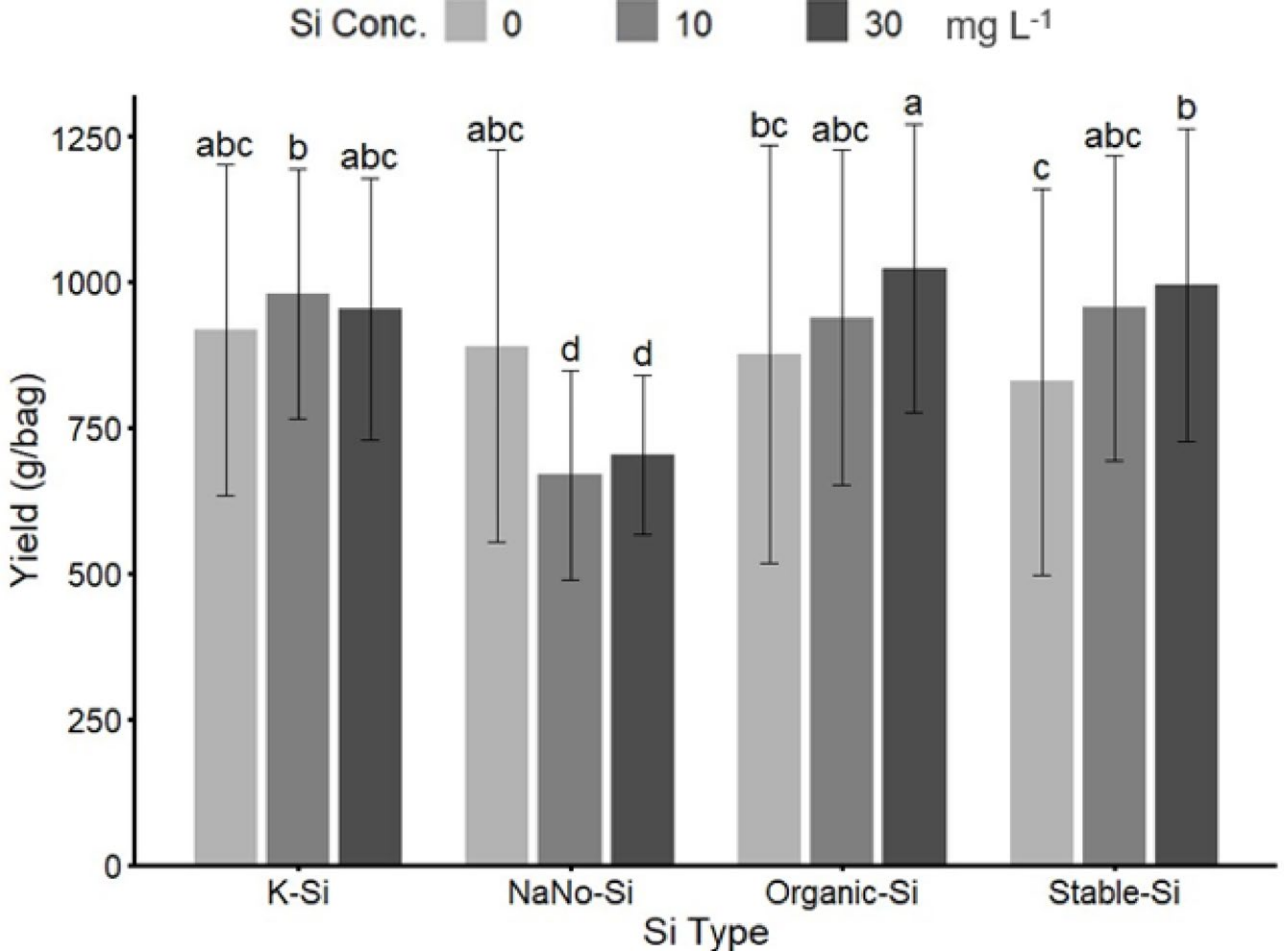
Effect of different types (K-Si, NaNo-Si, Organic-Si) and rates (0, 10, and 30 mg L^− 1^) of Si fertilizers on Strawberry Yield. Bars sharing the same letter are not significantly different at *p* ≤ 0.05 (LSD test).

The mean comparison results (Fig. 4) showed that organic silica treatment at 30 mg L^−1^ produced the highest strawberry yield which was approximately 25–30% greater than yields from nano-silica at the same rate. In contrast, nano silica treatments at 10 and 30 mg L^− 1^ resulted in the lowest yields, up to 20–25% lower than the top-performing treatments (Fig. 4). Potassium silicate and stable silicon fertilizers at 10 and 30 mg L^−1^ also performed well with yields not significantly different from organic silicon. Overall, organic silicon fertilizers delivered the best performance (Fig. 4).

#### Effect of salinity levels and different rates or types of Si fertilizers

**Fig. 5:**
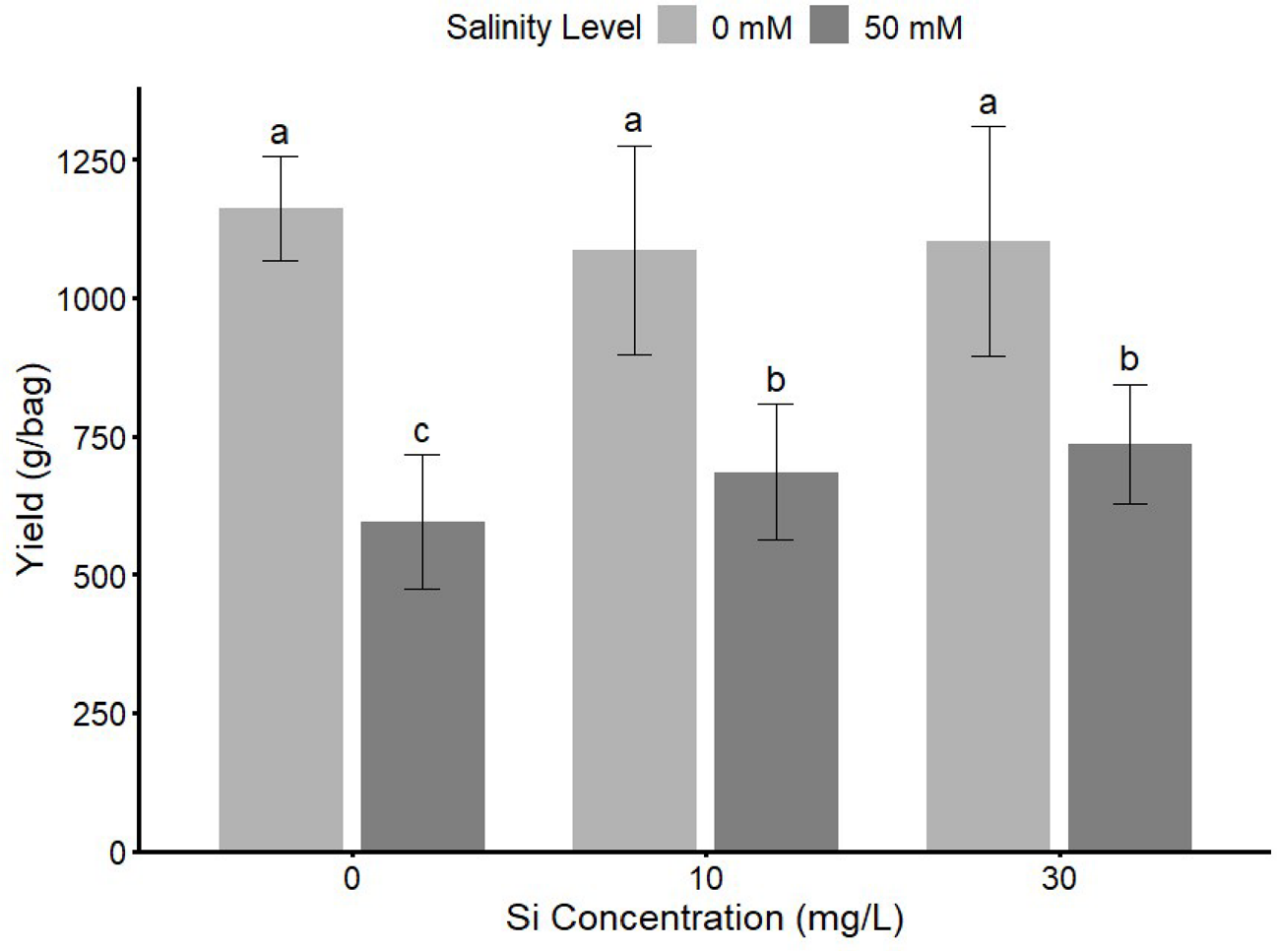
Effect of different rates (0, 10, and 30 mg L^−1^) of Si fertilizers and salinity levels (0 and 50 mM) on Strawberry Yield. Bars sharing the same letter are not significantly different at *p* ≤ 0.05 (LSD test).

**Fig. 6:**
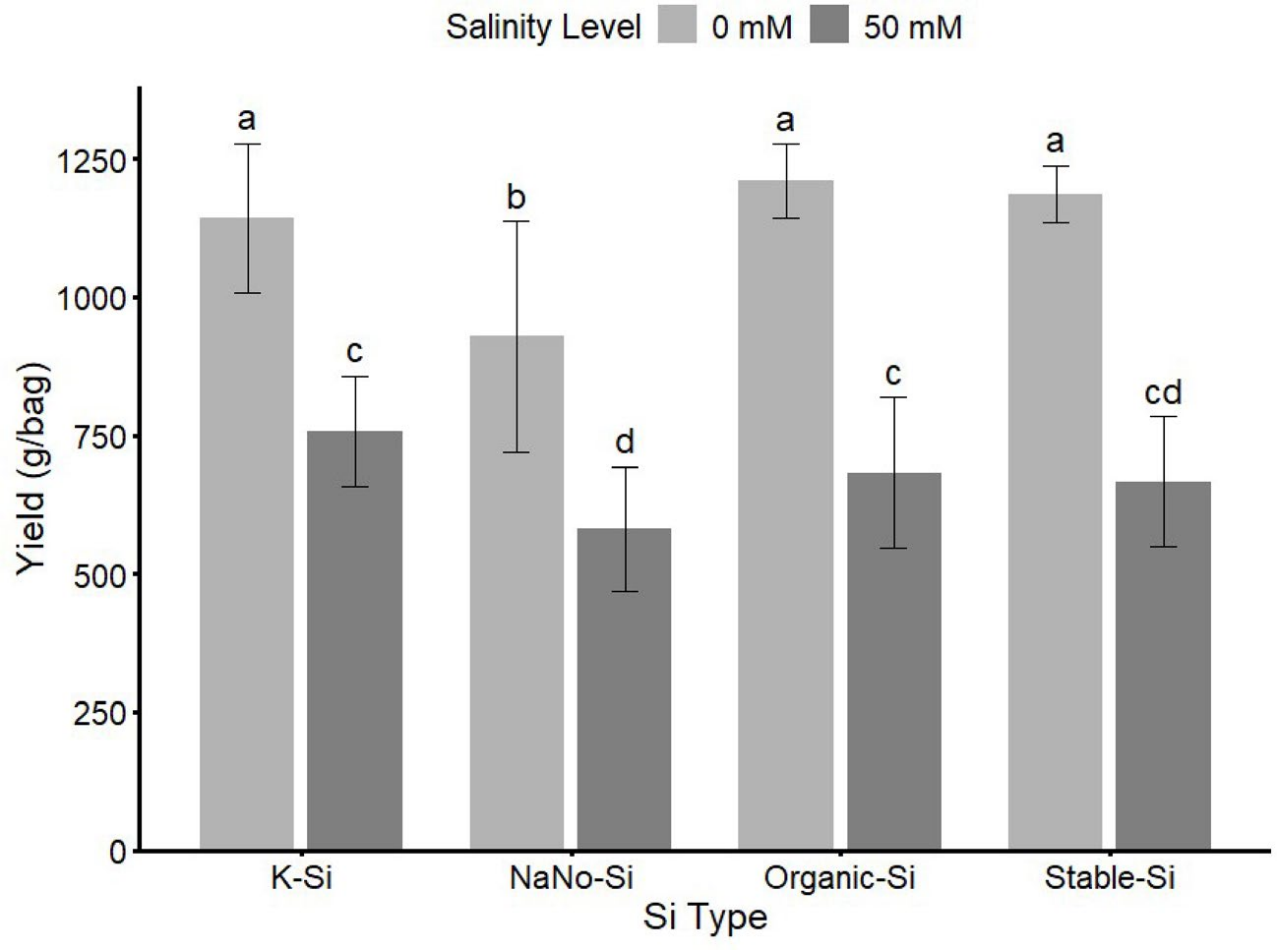
Effect of different types (K-Si, NaNo-Si, Organic-Si) of Si fertilizers and salinity levels on Strawberry Yield. Bars sharing the same letter are not significantly different at *p* ≤ 0.05 (LSD test).

The results presented in Figs. 5 and 6 showed Si rates and types both affected strawberry yield under saline and non-saline conditions. In non-saline conditions (zero mM NaCl), Si concentration increase had high yields in all treatments. However, under salinity stress (50 mM NaCl), yields decreased significantly. Organic Si and K-Si under salinity maintained comparatively better performance.

### Correlation matrix

**Fig. 7:**
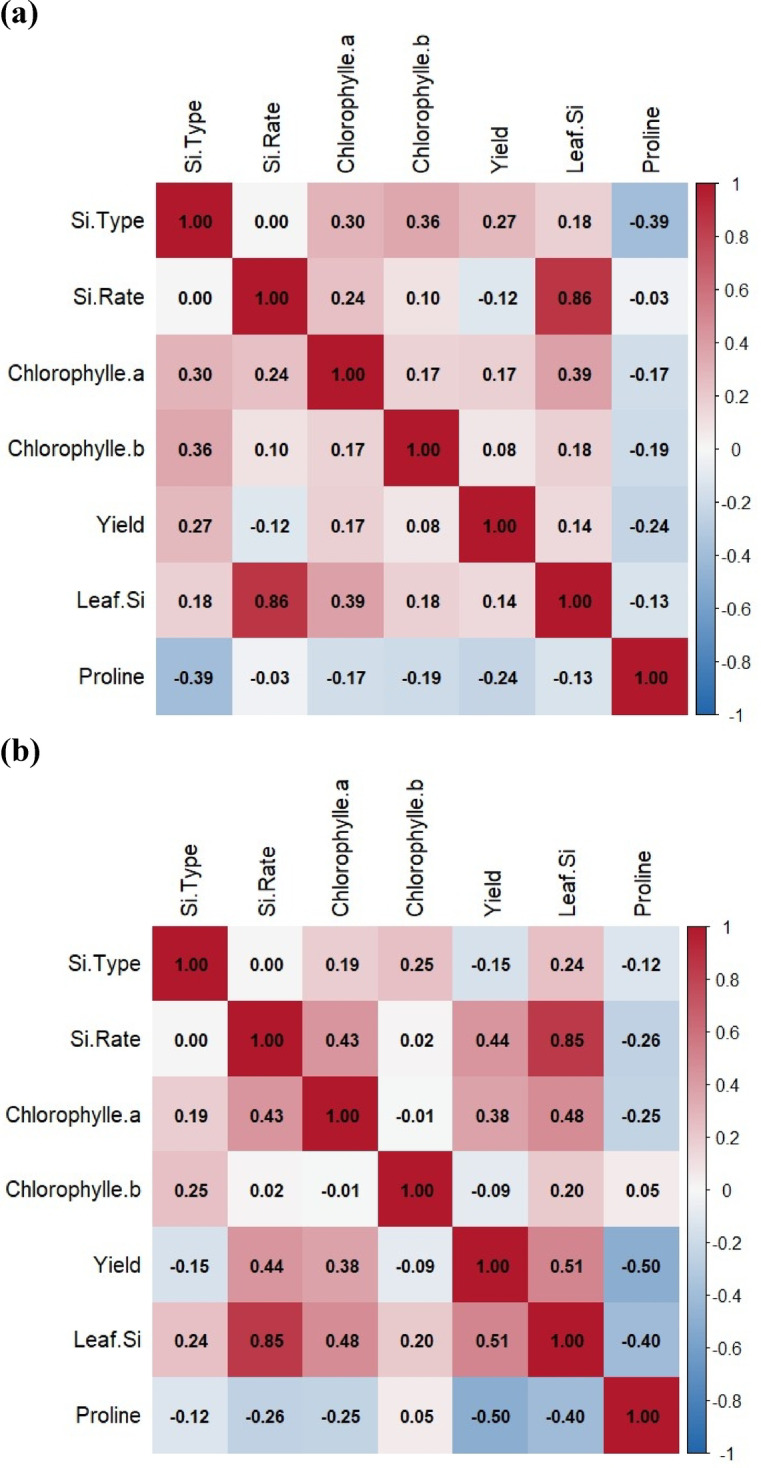
Correlation matrix between different measured factors in (**a**) non-saline condition (0 mM) and (**b**) salinity condition (50 mM).

Correlation analyses are presented in Fig. 7 under non-saline and saline conditions. Under non-saline conditions (Fig. 7a), both Si type and Si application rate showed positive correlations with chlorophyll a (*r* = 0.30 and 0.24, respectively) and chlorophyll b (*r* = 0.36 and 0.17, respectively). These associations indicate that more soluble or bioavailable forms of Si, as well as higher application doses. Moreover, yield exhibited moderate positive correlations with chlorophyll a (*r* = 0.39) and chlorophyll b (*r* = 0.18) (Fig. 7a). It suggests that increases in photosynthetic pigment levels translate into improved productivity when plants are not stressed. Proline showed negative correlations with Si-related variables and chlorophyll pigments (e.g., *r* = − 0.39 with Si type and − 0.17 with chlorophyll a (Fig. 7a). It reflects Proline typical role as a stress indicator; plants with lower stress (higher Si uptake and pigment levels) accumulated less proline. These relationships illustrate Si contributes to improve physiological function by supporting pigment stability and reducing the need for osmotic adjustment. In contrast, under saline conditions (Fig. 7b), the correlation between Si type and chlorophyll a dropped to *r* = 0.15, and to chlorophyll b to *r* = 0.22. The correlations between yield and both Si rate and chlorophyll pigments deteriorated or even turned slightly negative (Fig. 7b). It indicates under salinity conditions; increased pigment content does not necessarily translate to higher yields due to the physiological restrictions imposed by salt stress. In addition, the relationship between leaf Si and yield turned negative (*r* = -0.50). Proline under salt stress showed no clear relationship with the other factors (from − 0.12 to 0.10) (Fig. 7b). Overall, the correlation patterns indicate that under optimal conditions, Si enhances chlorophyll retention and yield. However, under salinity, Si still contributes to pigment stabilization and reduced stress signaling (proline).

### VNIR spectroscopy

**Fig. 8:**
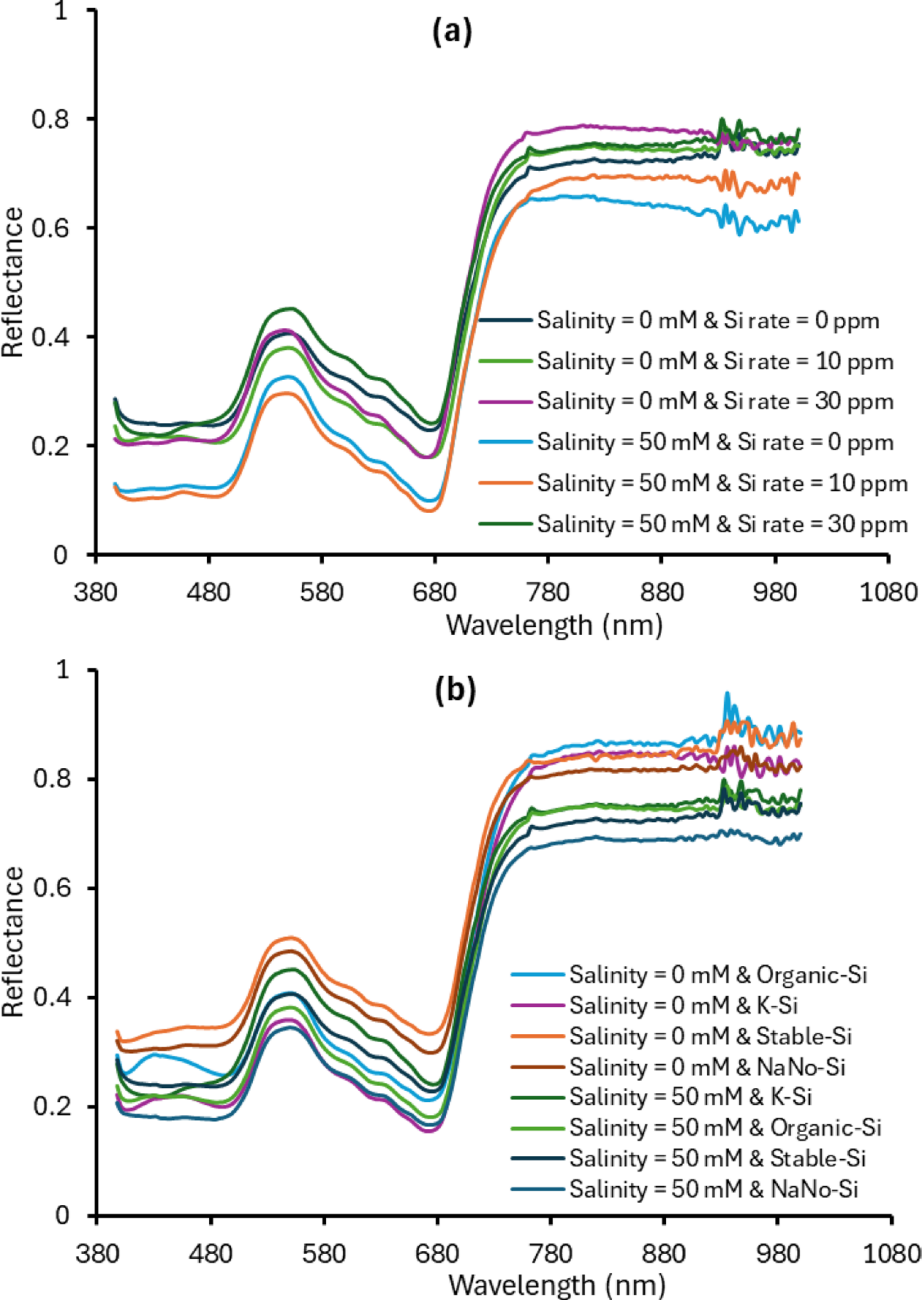
Mean spectral reference of plant leaf for different salinity level, Si rate, and Si type.

Figure 8 shows the mean spectral reflectance of strawberry leaves under different salinity levels, Si rates, and Si types. Under non-saline conditions (0 mM), plants had higher reflectance (Fig. 8). Under saline conditions (50 mM), reflectance was lower overall, but adding Si, especially at 30 mg Si L^−1^, helped reduce the negative effects of salt stress on the leaves’ spectral response (Fig. 8). Additionally, organic Si led to the higher reflectance under both normal and salt-stressed conditions Fig. 8). Therefore, it helped keep the leaves healthier and better at absorbing light. The results show that organic Si was the best at supporting leaf health and increase the plants tolerance for salt stress.

**Fig. 9:**
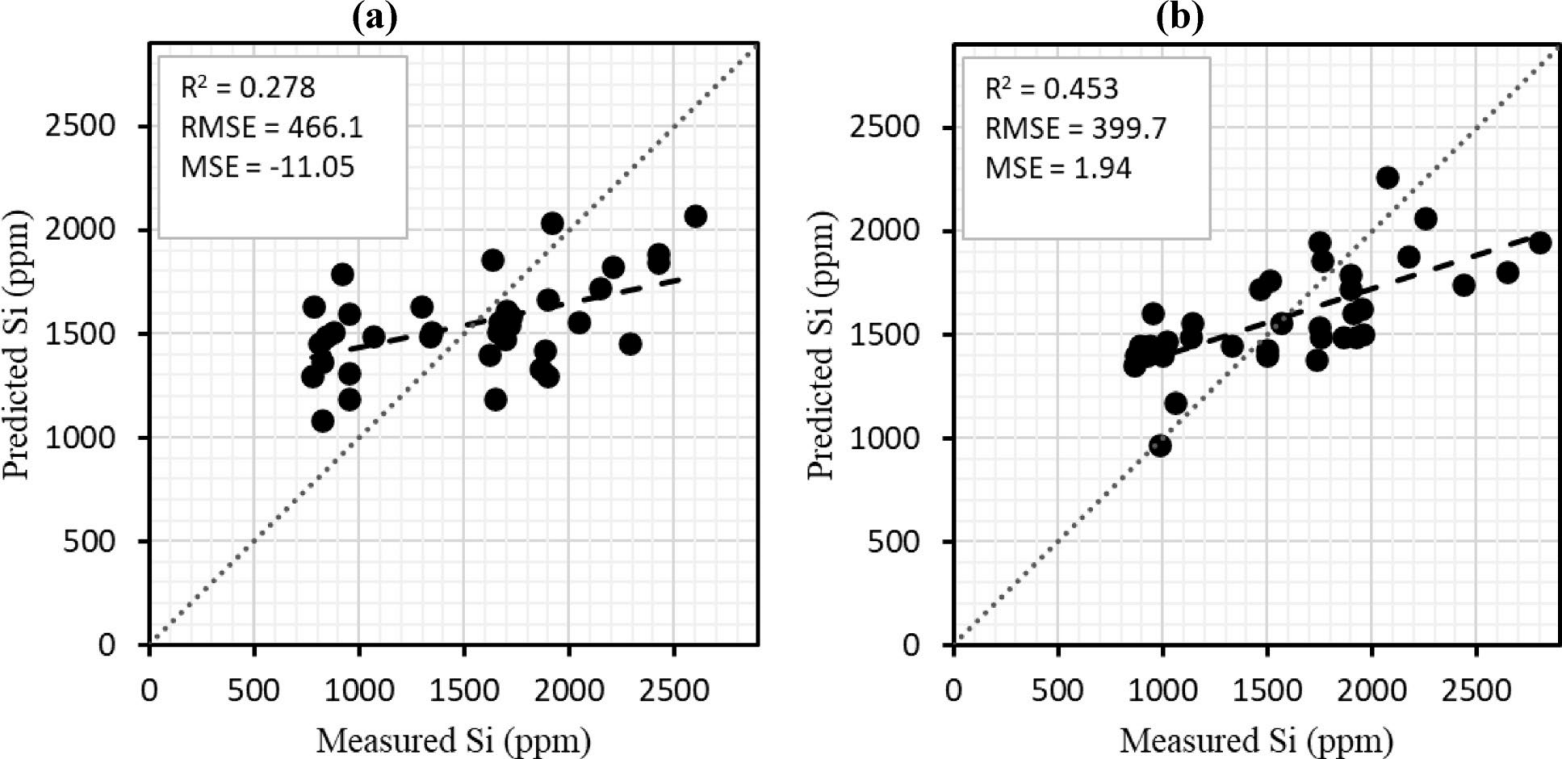
VNIR spectroscopy coupled with the PLSR method for predicting leaf Si in non-saline (**a**) and saline (**b**) conditions.

Figure 9 shows the results of PLSR method combined with VNIR data to predict leaf Si content under non-saline (a) and saline (b) conditions. Under non-saline conditions, the model yield a R² value of 0.278 with RMSE = 466.1 and MSE = − 11.05 (Fig. 9). However, under saline conditions, the model performed better with a R² = 0.453, RMSE = 399.7, and MSE = 1.94 (Fig. 9). However, these R² values reflect only weak-to-moderate predictive ability, and caution is required in interpreting the precision of these models. At this stage, the results should be considered preliminary and highlighting the potential of VNIR–PLSR as a proof-of-concept rather than a robust predictive tool. Additionally, we suggest that future improvements could be achieved by enlarging the dataset, applying advanced machine learning algorithms, and integrating additional spectral or physiological parameters to enhance robustness and accuracy.

## Discussion

In this section, we interpret the physiological, biochemical, and yield responses observed in strawberry plants under salinity and silicon treatments. It links these findings to underlying mechanisms and comparing them with results from previous studies to provide broader scientific context. These findings are consistent with earlier reports that strawberries are highly sensitive to salinity^3,5^. Under 50 mM NaCl, reductions in chlorophyll pigments and yield were observed, while proline accumulation increased. It confirms its role as a biochemical marker of salt-induced osmotic stress.

Among the four Si sources tested, stabilized silicic acid and potassium silicate were the most effective in mitigating salt stress. They significantly enhanced chlorophyll contents, reduced oxidative damage, and improved yield under salinity stress. In contrast, nano-silica and organic silica showed weaker effects. This may be related to slower mobility or lower efficiency of uptake through leaf cuticles. These findings support the notion that the chemical form of Si influences its effectiveness in salt stress mitigation. Similarly, the present study showed that organic silica at 30 mg L⁻¹ significantly improved chlorophyll b and increased plant yield under salinity stress. This is consistent with other studies^36^. Epstein^36^ highlighted that Si improves nutrient uptake and promotes stronger photosynthetic systems. The positive effects of Si fertilizers under salt stress can be explained by their role in reducing osmotic stress, maintaining photosynthesis, and improving ionic balance^37,38^. Yaghubi et al.^38^ also demonstrated that potassium silicate recovered yield under salinity on strawberry cultivars.

Several mechanisms may explain the observed improvements. First, Si is known to reduce Na⁺ uptake and transport^39^. Second, Si enhances antioxidant enzyme activity that helps reduce reactive oxygen species^40^. The higher chlorophyll a and b contents in Si-treated plants suggest improved photosynthetic efficiency and pigment protection. Third, Si may contribute to osmotic adjustment by regulating water status and reducing membrane permeability. In this study, the lower proline levels in Si-treated plants compared to untreated controls suggest that plants experienced less oxidative and osmotic stress when supplemented with Si. Such improvements may also result from enhanced nutrient availability and better physiological performance of plants under Si treatments as supported by previous studies on strawberry cultivars^38,41^. These results are in agreement with studies such as Li et al.^41^. Moreover, Si helps by enhancing biomass, root length, nutrient availability, improving water balance, boosting photosynthesis and carbohydrate production, and maintaining ionic balance^26,37^. Salt stress, on the other hand, imposes osmotic pressure, suppresses photosynthesis, and reduces carbohydrate synthesis^42^. Previous studies on strawberry cultivars such as Elsanta, Corona, and Selva have shown that salinity reduces yield by suppressing leaf area and leaf number^3,43^. Yaghubi et al.^38^ reported that potassium silicate reduces the negative effects of salinity on strawberry varieties grown in soilless culture. Orsini et al.^43^ reported strawberry yield reduction under salt stress due to reduced leaf area and fewer leaves. Savvas et al.^37^ showed that Si treatment in hydroponic zucchini squash relieved the injurious effects of salt stress on yield.

In strawberries, Ferreira et al.^5^ observed that high Si supply under saline irrigation improved plant survival but did not consistently increase fruit yield. It suggests that physiological protection may come at the expense of reproductive output when stress is severe. Likewise, in cucumbers and tomatoes, excessive foliar Si deposition was shown to saturate leaf tissues, where further accumulation no longer benefitted photosynthesis but diverted energy and resources away from fruit development^37^. Moreover, studies in tomato and other vegetables^44–46^ have shown similar trends. Si application or seed treatments alleviate NaCl-induced damage by stabilizing photosynthetic pigments, enhancing antioxidant activity, and maintaining ionic homeostasis. For example, Habibi et al.^44,47^ reported that tomato plants exposed to salinity showed improved chlorophyll retention, reduced oxidative stress, and enhanced K⁺/Na⁺ ratio when supplemented with Si-based treatments.

VNIR spectroscopy coupled with PLSR demonstrated only weak-to-moderate predictive power (R² = 0.278–0.453) for estimating leaf Si content. These results should be considered preliminary, serving as a proof-of-concept rather than a robust predictive tool. Nonetheless, the ability to detect moderate associations indicates potential for developing non-destructive monitoring strategies. Future improvements may come from increasing sample size, applying advanced machine learning methods, and integrating additional physiological traits or spectral features to strengthen predictive accuracy.

## Conclusion

This study demonstrated that stabilized silicic acid and potassium silicate, especially at 30 mg L^−1^, were the most effective Si sources for improving strawberry tolerance to salinity. These treatments enhanced chlorophyll content, reduced oxidative stress indicators, and helped sustain yield under 50 mM NaCl stress, confirming their value as practical tools for managing salt-affected production systems. Although VNIR–PLSR modeling showed only weak-to-moderate predictive power for leaf Si content, the preliminary results indicate potential for future development of non-destructive monitoring approaches. From an agronomic perspective, foliar Si applications represent an accessible and environmentally safe strategy that growers can integrate into existing management practices to help mitigate salinity damage. Future work should validate these findings under field conditions and explore long-term and cultivar-specific responses, as well as the underlying physiological and molecular mechanisms of Si-mediated stress tolerance.

## Data Availability

The data that support the findings of this study are available from the corresponding author, upon reasonable request.
